# Systematic investigation of the impact of screw elements in continuous wet granulation

**DOI:** 10.1016/j.ijpx.2024.100273

**Published:** 2024-08-08

**Authors:** Katharina Kiricenko, Robin Meier, Peter Kleinebudde

**Affiliations:** a Heinrich Heine University Düsseldorf, Faculty of Mathematics and Natural Sciences, Institute of Pharmaceutics and Biopharmaceutics, Universitätsstrasse 1, 40225 Düsseldorf, Germany; b L.B. Bohle Maschinen und Verfahren GmbH, 59320 Ennigerloh, Germany

**Keywords:** Continuous manufacturing, Twin-screw wet granulation, Screw configuration, Design of experiments

## Abstract

Twin-screw wet granulation (TSG) is a continuous manufacturing technique either for granules as final dosage form or as an intermediate before tableting or capsule filling. A comprehensive process understanding is required to implement TSG, considering various parameters influencing granule and tablet quality. This study investigates the impact of screw configuration on granule properties followed by tableting, using a systematic approach for lactose-microcrystalline cellulose (lactose-MCC) and ibuprofen-mannitol (IBU) formulations. The most affecting factor, as observed by other researchers, was the L/S ratio impacting the granule size, strength and tabletability. Introducing tooth-mixing-elements at the end of the screw, as for the IBU formulation, resulted in a high proportion of oversized granules, with values between 36% and 78%. Increasing the thickness of kneading elements (KEs) produced denser, less friable granules with reduced tablet tensile strength. Granulation with more KEs, larger thickness or stagger angle increased torque values and residence time from 30 to 65 s. Generally, IBU granules exhibited high tabletability, requiring low compression pressure for sufficient tensile strength. At a compression pressure of 50 MPa, IBU tablets where at least one kneading zone was included resulted in approximately 2.5 MPa compared to lactose-MCC with 0.5 MPa. In conclusion, the TSG process demonstrated robustness by varying the screw design with minimal impact on subsequent tableting processes.

## Highlights

• Screw configuration analysis via DoEs for two formulations revealed L/S ratio as the most influential parameter during TSG.
• TMEs caused more oversized granules in the IBU formulation due to longer residence time and increased granule growth.
• At least one kneading zone, independent of type, is required to produce granules and tablets with sufficient strength.

## Graphical Abstract

**Figure:**
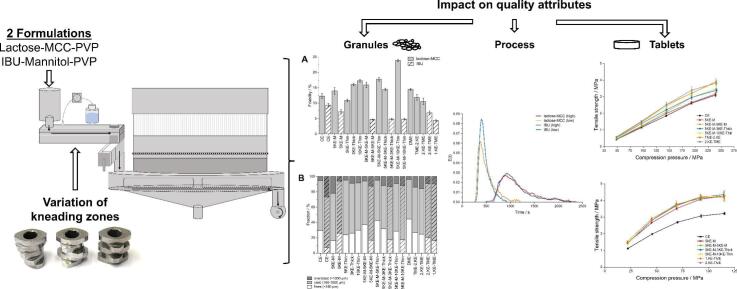

## 1 Introduction

Granules are frequently used either as intermediates prior to tableting, capsule filling or as the final oral dosage form. Consequently, wet granulation emerges as the most used technique in the manufacturing of solid oral dosage forms (Leane et al., 2018). Various wet granulation techniques exist, such as high-shear granulation, twin-screw wet granulation (TSG) and fluidized bed granulation (FBD) (Arndt et al., 2018). The choice of granulation technique significantly impacts the properties of both the granules and the resulting tablets. Arndt et al. compared the granule strength and tablet tensile strength (TS) of one formulation produced using these three methods. High-shear granulation yielded the highest granule strength due to its greater densification during granulation, resulting in the lowest tablet TS. Conversely, FBD led to the highest tabletability. This study demonstrated comparable results between FBD and TSG for the investigated formulation (Arndt et al., 2018). Furthermore, one advantage of the TSG technique is its capability for continuous manufacturing. Extensive research has been conducted in the past to understand the TSG process and the influence on granules and tablet attributes. Various process parameters and formulation properties influence the characteristics of granules and the resulting tablets.

One influential factor in the granulation process is the screw configuration, which may consist of different possible elements such as conveying elements (CEs), kneading elements (KEs) and size reducing elements. The arrangement and characteristics of these elements significantly influence the process and the properties of granules. For example, Djuric and Kleinebudde observed that the flight pitch of CEs impacted the fraction of fines and oversized granules. The use of only CEs resulted in granules with high porosity (Djuric and Kleinebudde, 2008; Peeters et al., 2023) and high amount of fines with a bimodal granule size distribution (GSD) (Thompson and Sun, 2010). KEs, on the other hand, serve as mixing and shearing zones affecting granule shape and liquid distribution efficiency, thereby influencing GSD (El Hagrasy and Litster, 2013; Peeters et al., 2023; Thompson and Sun, 2010; Vercruysse et al., 2015b). Increasing the number of KEs enhanced granule growth and reduced the fraction of fines (Portier et al., 2020a; Vercruysse et al., 2015a), while also reducing the porosity of granules (Peeters et al., 2023) due to increased densification, potentially leading to reduced tablet TS (Djuric and Kleinebudde, 2008; Kashani Rahimi et al., 2020).

A few systematic investigations using a design of experiments (DoEs) are available that consider the impact of screw configuration on granules (Kumar et al., 2016a; Portier et al., 2020a; Vercruysse et al., 2012) and on tablets (Vercruysse et al., 2012). In these studies, either the number of KEs and angle were varied, or the fraction of kneading zones and thickness. None of these studies covered all three variations in the KE zone, number, thickness, and offset angle, which were investigated in this study.

Liquid-to-solid (L/S) ratio is the most influential thus critical process parameter in wet granulation. Increasing L/S ratio resulted in increased granule size, oversized and reduced fraction of fines (Ito and Kleinebudde, 2019; Meng et al., 2019; Osorio et al., 2017; Portier et al., 2020b; Stauffer et al., 2019). Further parameters, such as throughput (Dhenge et al., 2010; Kumar et al., 2016a; Li et al., 2014), screw speed (Kumar et al., 2016a; Mundozah et al., 2020; Peeters et al., 2023) and barrel temperature (Meng et al., 2019; Stauffer et al., 2020; Vanhoorne et al., 2016b) also affect the granule and tablet attributes. Beside process parameters, formulation properties also influence the granulation process and the properties of granules and tablets (Fonteyne et al., 2015; Meier et al., 2015; Vandevivere et al., 2022).

The aim of the current study was to further understand the effect of the screw configuration, particularly the kneading zone, including possible variables such as the number of KEs, offset angle, and thickness of the KEs, and to identify possible interactions between the factors of KE and L/S ratio during TSG on granule and tablet attributes. Additionally, the influence of varying kneading zones, either one or two, with or without mixing elements, was investigated regarding the impact on GSD, granule friability and tabletability, compactability and compressability.

## 2 Materials and methods

### 2.1 Materials

Two formulations were utilized to investigate the screw configuration in wet granulation. The first formulation comprised 80.0% (w/w) alpha-lactose monohydrate (GranuLac® 200, MEGGLE; Wasserburg am Inn, Germany), 17.0% (w/w) microcrystalline cellulose (MCC, VIVAPUR® 101, JRS PHARMA, Rosenberg, Germany) and 3.0% (w/w) Polyvinylpyrrolidone K 30 (PVP, Kollidon® 30, BASF SE, Ludwigshafen, Germany) as a binder. This resulted in a formulation with good solubility, including MCC as a high water absorption excipient, allowing for the use of broader L/S ratios. The second formulation contained 48.5% (w/w) ibuprofen (IBU, Ibuprofen 50, BASF SE, Ludwigshafen, Germany) as active pharmaceutical ingredient (API), 48.5% (w/w) mannitol (Pearlitol® 200 SD, Roquette, Lestrem, France) and 3.0% (w/w) PVP K 30. The second formulation contains 48.5% of a hydrophobic API, which belongs to Class II of the Biopharmaceutical Classification System (BCS) due to its low solubility (Han et al., 2023). Therefore, this formulation is expected to present more challenges in achieving sufficient liquid distribution to improve granule and tablet quality and to be more sensitive in changes of L/S ratio. Demineralized water was used as granulation liquid. Iron (III) oxide (Carl Roth, Karlsruhe, Germany) was used as a tracer for residence time distribution measurements. For tableting, 1.0% (w/w) magnesium stearate (MgSt, Parteck® LUB MST, Merck, Darmstadt, Germany) was applied as a lubricant.

### 2.2 Methods

#### 2.2.1 Preparation of powder mixtures

Before blending, IBU was deagglomerated using a high-speed conical mill (BTS 100, L.B. Bohle Maschinen und Verfahren, Ennigerloh, Germany) at 300 rpm provided with a sieve mesh of 1.0 mm. 25 kg of each formulation mixture was blended for 20 min at 25 rpm in a lab-scale blender (LM 40, L.B. Bohle Maschinen und Verfahren, Ennigerloh, Germany).

#### 2.2.2 Continuous granulation and drying

TSG and drying were examined using a QbCon® 1 (L.B. Bohle, Maschinen und Verfahren, Ennigerloh, Germany). The QbCon® 1 includes a powder feeder, a TSG with a screw diameter (D) of 16 mm and a vibrated fluidized bed dryer. Powder feeding was performed using a loss-in weight feeder (DIW-PE-GZD-P 150.12 Gericke, Regensdorf, Switzerland) and liquid feeding using a micro-gear pump (MZR-7205, HNP-Mikrosysteme GmbH, Schwerin, Germany) equipped with a nozzle diameter of 0.12 mm. Liquid addition occurred directly before the first kneading block. Powder feed rate of 1.2 kg/h, screw speed of 100 rpm and barrel temperature of approximately 25 °C were kept constant for all experiments. Torque was recorded during the process at a frequency of 1 Hz. The L/S ratio was adjusted dependent on the experiment and formulation.

**Fig. 1:**
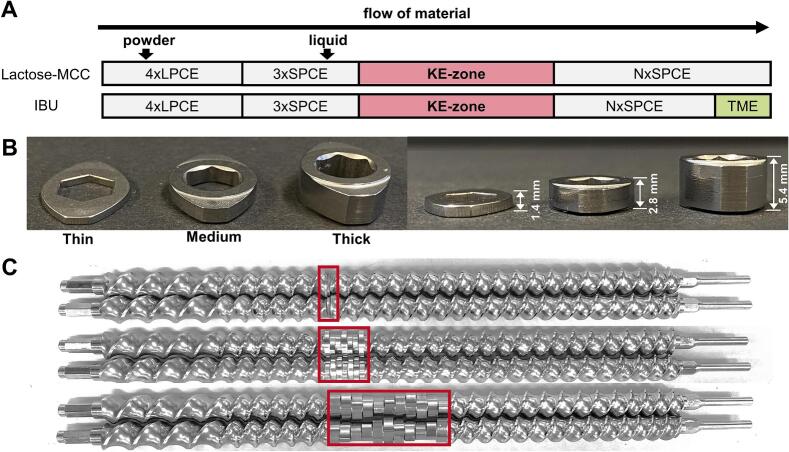
Screw configuration scheme applied for the DoE (A), pictures of the different KE (B) and exemplary screw configuration with different KE-zones (C) without TME.

Drying of lactose-MCC granules was carried out at a temperature of 75 °C with an air flow of 15 Nm^3^/h and a vibration acceleration of 5 m/s^2^. For IBU granules, the drying parameters were set to 60 °C, 20 Nm^3^/h and 5 m/s^2^. After residence time measurements, 600 g of granules were collected at the outlet of the dryer. The screw configuration comprised long pitch conveying elements (LPCEs) with a screw length of 1D which were positioned always at the beginning to facilitate the forward transport of powder and prevent accumulation. Further, short pitch conveying elements (SPCEs) with lengths of either 1D or 1.25D were applied depending on the kneading zone. The use of KEs varied across experiments, involving different stagger angles, thickness and number of KEs (Fig. 1).

#### 2.2.3 Design of experiments

A full factorial DoE was performed to assess the influence of the screw configuration and L/S ratio on the granulation process as well as on the properties of granules and tablet (Table S1). The DoE was examined with different L/S ratios for the lactose-MCC (15% - 25%) and IBU (13.5–17.5%) formulation. Besides L/S, the number (3−11), thickness (1.4–5.4 mm) and stagger angle (30° - 90°) of the KEs were investigated as factors. For that, the process was halted and both the barrel and screws were cleaned before switching to a different screw configuration. In total, 16 runs with additional 3 center points were conducted in randomized order for each formulation. The DoE setup and evaluation were performed using MODDE Pro (V13.0.2, Sartorius Stedim Data Analytics AB, Malmö, Sweden). Models obtained were optimized through backward regression by eliminating non-significant effects based on the *p*-value >0.05. The experimental design is listed in supplement Table S1. During the granulation of MCC-lactose, the low L/S of 15% could not be set using KE-zone of thick, eleven KEs with a stagger angle of 90° and thus L/S was adjusted to the lowest possible L/S ratio of 21.5%. At L/S of 15%, the torque exceeded the machine limit and the process was stopped. The center point for thickness was intended to be 3.4 mm but was adjusted to the available thickness of 2.8 mm and named as medium thickness (M). Using a thickness of 2.8 mm as the center point resulted in an asymmetrical design. Schematic representations of the applied screw configurations are shown for each formulation in Fig. 1A by adjusting the number (N) of SPCEs, dependent on the KE-zone. For the granulation of the IBU formulation, one tooth-mixing-element (TME) with a screw length of 1D was utilized at the end of the screw configuration. The applied KE discs of varying thicknesses are illustrated in Fig. 1B. Fig. 1C represents three examples of screw configurations for the lactose-MCC formulation, demonstrating the length of the KE-zone where three thin, seven normal and eleven thick KEs are shown.

#### 2.2.4 Systematic variation of kneading zone

**Fig. 2:**
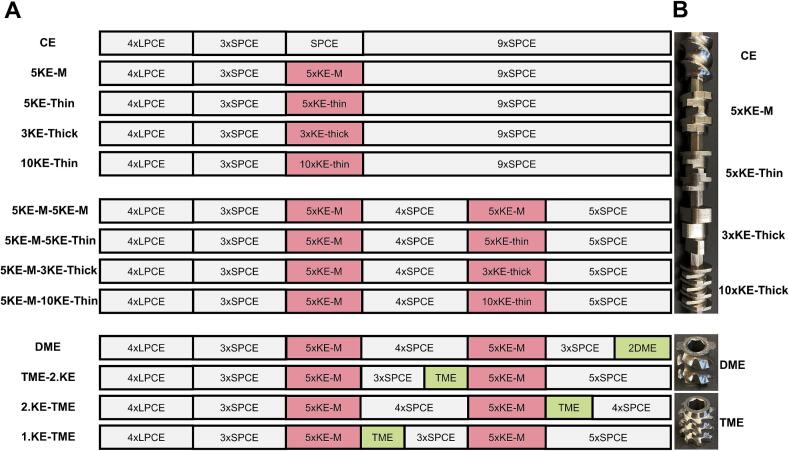
Screw configurations scheme (A) and pictures of the different elements (B).

Another approach for varying the KE zone was to keep the length of 1D constant independent of the applied thickness of the KE. Therefore, either only CEs were used, or one or two KE-zones were investigated. A stagger angle of 60° was kept constant for the investigated configurations. Fig. 2A displays the applied configurations. Except for one configuration (1.KE-TME), all other configurations were conducted with lactose-MCC formulation. Additionally, the following configurations were investigated using the IBU formulation: CE; 5KE-M; 5KE-M-5KE-M; 5KE-M-3KE-Thick; 5KE-M-10KE-Thin; 2.KE-TME and 1.KE-TME. The configuration using distributive mixing element (DME) was only applied with lactose-MCC. The region after the second KE-zone consisted of different lengths of SPCEs, either 1.25D or 1D, to keep the total screw length as close as possible to 320 mm. When using only one KE-zone, SPCE with 1.25D were applied. In case of 5xSPCE at the end of the screw configuration, this consisted of 2x1.25D and 3x1D SPCEs. Fig. 2B illustrates each KE-zone. The granulation and drying parameters for each formulation are mentioned in the section 2.2.2. *Continuous granulation and drying.*

#### 2.2.5 Residence time distribution

Residence time distribution (RTD) was measured inline during granulation and evaluated afterwards using a camera based ExtruVis3 system and software (ExtruVis, MeltPrep, Graz, Austria). 10–15 mg of Iron (III) oxide was added as a pulse at the powder addition port. The camera was positioned at the beginning of the dryer in front of a window. At this position, the granules enter the dryer. A fivefold measurement was conducted. The resulting curve was smoothed and normalized to the area under the curve by the software for evaluation. This yielded an exit age function E(t). For comparison, the mean residence time (MRT) was utilized.

#### 2.2.6 Loss-on-drying determination

The loss-on-drying (LOD) was determined in triplicates offline with a moisture analyzer (MA 100, Sartorius, Göttingen, Germany) using a sample size of approximately 2 g of dried granules. The granules were dried at 80 °C (lactose-MCC granules) or 60 °C (IBU granules) using a termination criterion set at 0.1% of the mass difference within 150 s.

#### 2.2.7 Sample preparation

Sampled granules were divided using a sample divider (Sample divider PT 100, Retsch, Haan, Germany) into appropriate sizes before subsequent measurement.

#### 2.2.8 Granule size distribution

Three sub-samples of approximately 10 g each were measured using dynamic image analysis (Camsizer XT, Retsch, Haan, Germany) in X jet mode. A dispersion pressure of 0.3 bar was applied for each measurement. The x_c min_ diameter was utilized for GSD resulting in three quantiles of the volume distribution (Q3): x10, x50 and x90. Here, x_c min_ diameter indicates the shortest chord among the maximum chords x_c_ measured. Each sample was fractionated using vibrating sieve analysis (AS control, Retsch, Haan, Germany). The granules were sieved for 10 min at an amplitude of 1 mm using sieves with mesh size of 1000, 800, 500, 355, 250 and 180 μm. Fraction of granules below 180 μm were classified as fines, while those above 1000 μm as oversized. Thus, the fraction between 180 μm to 1000 μm was designated as yield and was used for friability and tableting.

#### 2.2.9 Granule friability

The granule friability was determined for the fraction between 180 and 1000 μm in triplicate samples of approximately 10 g each, using an air jet sieve (LS-N 200, Hosokawa Alpine, Augsburg, Germany) with a sieve size of 125 μm. The measurements were conducted according to Duric and Kleinebudde (Djuric and Kleinebudde, 2008).

#### 2.2.10 Tableting

The yield fraction was blended for 3 min with 1.0% MgSt using a turbula mixer (T2C, Willy Bachofen, Muttenz, Switzerland). Tableting was carried out using a STYL'One Evolution (Medelpharm, Beynost, France) equipped with 11.28 mm flat-faced Euro-D punches. Ten tablets of 300 mg each were produced at five compression pressures by manually filling the die due to limited material availability. Lactose-MCC granules were compressed at 50, 100, 150, 200 and 250 MPa while IBU granules were compressed at 25, 50, 75, 100 and 125 MPa.

#### 2.2.11 Characterization of tablets

Tablets were stored at 21 °C and 45% relative humidity for at least 48 h before characterization. The diameter, thickness, weight and crushing force of ten lactose-MCC tablets were measured using an automatic tablet tester (Smart Test 50, Pharmatron Dr. Schleuniger, Thun, Switzerland). IBU tablets could not be measured at all five compression pressures using the automatic tablet tester due to overload error indication. Therefore, the dimensions of the six tablets were measured manually using an electronic micrometer (Mitutoyo Corporation, Kawasaki, Japan). The crushing force of the IBU tablets was determined with a tablet hardness tester (TBH 210, ERWEKA, Langen, Germany). For all tablets, the TS was calculated according to Fell and Newton (Fell and Newton, 1970). The solid fraction of the tablets was also investigated and calculated as described by Köster and Kleinebudde (Koster and Kleinebudde, 2023). For this, the tablet density was determined using the tablet dimensions and the weight. The required particle density of the primary powders was measured using helium pycnometry (AccuPyc 1330, micromeritics, Norcross, USA).

## 3 Results and discussion

### 3.1 Influencing factors – DoE

#### 3.1.1 Process

**Fig. 3:**
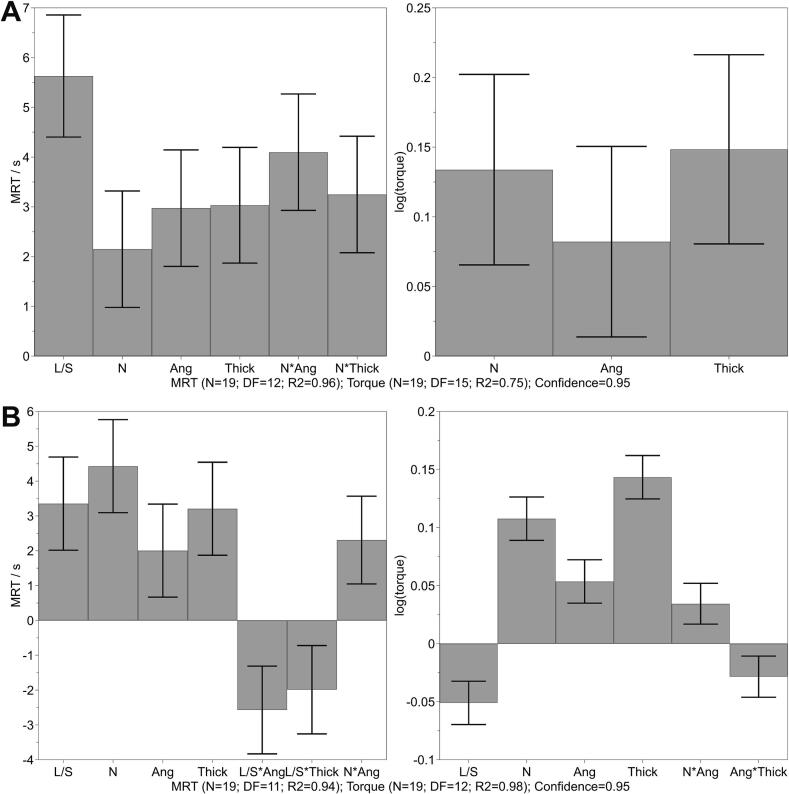
Coefficient plot for MRT (left) and logarithmic torque (right) obtained using lactose-MCC (A) and IBU (B) formulation, mean coefficient ± 95% confidence interval. The coefficient constants are 45.6 s for MRT and 0.97 for torque (A) and 48.5 s and 1.02 (B).

The influence on the wet granulation process was investigated using recorded torque values and MRT. Fig. 3 displays the coefficient plots of significant factors and interactions affecting MRT and torque. The obtained values for each run are listed in the supplement (Tables S2 and S3). The MRT values varied between 30.8 and 65.6 s for lactose-MCC and 31.4–63.1 s for IBU. MRT during granulation with lactose-MCC was primarily influenced by the L/S ratio, followed by number of KEs, angle and thickness of KEs (Fig. 3A). The interaction between the number of KEs and angle, as well as thickness, also had a significant positive influence. All factors exhibited a positive effect on MRT, for instance, an increase in L/S ratio resulted in a longer residence time. The increasing influence of L/S ratio (Kumar et al., 2016a; Liu et al., 2017; Shirazian et al., 2018) and the number of KEs (Kumar et al., 2016a) are in accordance with the literature. In comparison to the granulation of IBU, where TME was additionally positioned at the outlet of the screw configuration, the most affected factor was the number of KEs (Fig. 3B). Thereby, the interaction between L/S ratio and angle, and L/S and thickness of the KEs had a significantly negative impact. The usage of TME at the end of a screw configuration led to different interactions and different levels of coefficients for the same factors. Thus, the influence of the L/S ratio on the MRT is also formulation dependent. It seems that the analyzed L/S ratio values likely led to an increase in sluggishness of powder and flow restriction by applying higher stagger angle, resulting in increased residence time during granulation of lactose-MCC. An exemplary RTD curve is displayed in Supplement (Fig. S1), where all factors were set at both low and high level for both formulations. The MRT is not only increased by raising the factors but also the width of the distribution is broader, indicating the resistance to flow. Summary of fit of all investigated responses are attached in the supplement (Figs. S2 and S3).

The torque values ranged between 4.4 and 26.5 Nm during the granulation of lactose-MCC and 4.7–26.6 Nm using the IBU formulation. The highest torque was obtained when applying all factors at high level for lactose-MCC (Table S2). Therefore, the highest possible L/S ratio was used, otherwise, the torque exceeded the safety limit of 30 Nm. For the IBU formulation, the highest torque was achieved with a low L/S ratio while all other factors were at high levels (Table S3). The change in torque during granulation of lactose-MCC was positively correlated with the number of KEs, angle and thickness of the KEs. The increase in these factors resulted in an increase in the friction of granules against the wall and screws, indicated by the energy input of the motor (Kumar et al., 2016a; Liu et al., 2017). The L/S ratio had a negative impact on the torque, with increasing L/S ratio. However, when using lactose-MCC, this factor was not significant within the factor space. With an increasing L/S ratio, the granules are less exposed to friction and are easier transported with less energy input. This effect was observed for the granulation of IBU (Fig. 3B) and is consistent with the finding of Kumar et al. and Meng et al. (Kumar et al., 2016a; Meng et al., 2019). Contrary to this, the torque increased with increasing L/S ratio until over wetting occurred, at this point, the torque is decreased or become independent of the L/S ratio (Dhenge et al., 2010; Kumar et al., 2016b; Tu et al., 2013; Vanhoorne et al., 2016a). Tu et al. reported a fill level dependency, which is caused by different screw speed, thus affecting the impact of the L/S ratio on the torque (Tu et al., 2013). In this study, the screw speed was kept constant for all experiments at 100 rpm. This might be the reason why no effect of the L/S ratio was observed on the torque for the granulation of lactose-MCC or even a negative effect while granulation of IBU. Both formulations showed a significant positive effect of the screw configuration regarding number of KEs, thickness and stagger angle. In addition, significant interactions were obtained in the granulation of IBU between the number of KEs and angle, as well as angle and thickness of the KEs (Fig. 3B, right). The increased torque values by applying more KEs corresponds with the literature (Kumar et al., 2016a; Vanhoorne et al., 2016b; Vercruysse et al., 2012). The use of thick KEs with a stagger angle of 90° and the highest number (11 KEs) resulted in high torque values. The torque limit was even exceeded by using a low L/S ratio with this screw configuration. Therefore, during the granulation of lactose-MCC, the L/S ratio was set to the lowest possible value, which was 21.5% instead of 15%, as specified by the DoE.

#### 3.1.2 Granule properties

**Fig. 4:**
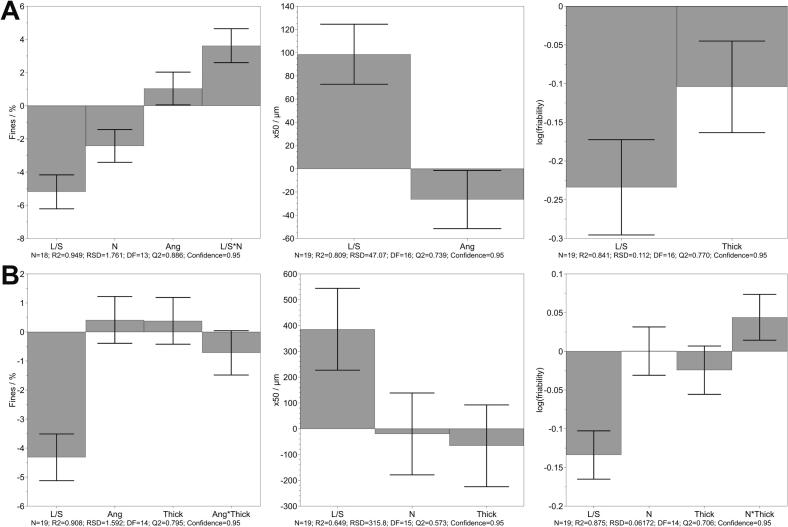
Coefficient plot for amount of fines (left), x50 value (middle) and logarithmic friability (right) obtained after granulation of lactose-MCC (A) and IBU (B) formulation, mean coefficient ± 95% confidence interval. The coefficient constants are 24.22% for fines, 170.68 μm for x50 and 1.18 for friability (A) and 7.43%, 1135.48 μm and 0.74 (B), respectively.

The influence of factors on the attributes of granules, including the amount of fines, granule size represented by x50 and friability were investigated and are displayed as coefficient plots in Fig. 4. The response variable, friability, was logarithmically transformed to achieve normally distributed values. The most influential factor in the chosen factor space, independent of the granule properties, was the L/S ratio, which is also reported by previous literature (Li et al., 2014; Meier et al., 2017; Osorio et al., 2017; Schmidt et al., 2016). Increasing L/S ratio resulted in decreased amount of fines and increased x50 value, as more liquid was available, leading to the formation of larger granules. Additionally, with increased L/S ratio, the residence time of granules inside the barrel increased, allowing more time for the dissolution of the binder PVP and formation of liquid bridges, consequently reducing granule friability. These finding contribute to Vandevivere et al., where lower friability was observed with increased L/S ratio due to greater liquid availability on the granule surface (Vandevivere et al., 2022).

During granulation of lactose-MCC, the amount of fines was affected not only by L/S ratio but also by the number of KEs and angle. Increasing the number of KEs in the screw configuration led to a reduction in fines, attributed to improved liquid distribution (El Hagrasy and Litster, 2013). Furthermore, the interaction between number of KEs and L/S ratio had a significant positive effect on the amount of fines. Increasing both resulted in reduced amount of fines. This observation was not found during granulation of IBU.

**Fig. 5:**
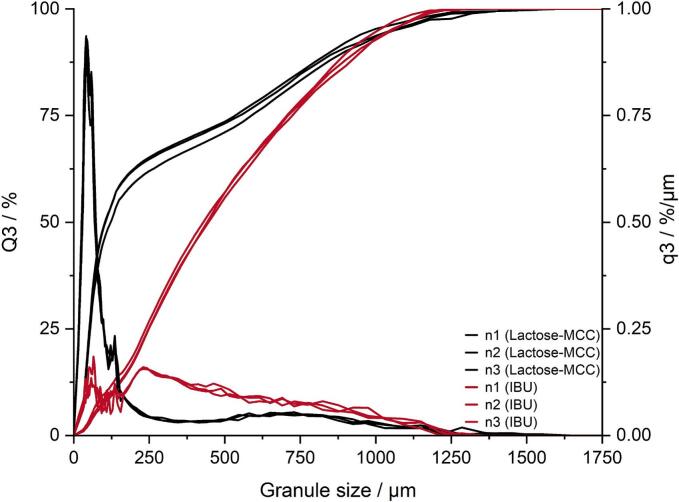
Cumulative GSD of granules displayed as single measurements produced at center point conditions during granulation of lactose-MCC (black) and IBU (red). (For interpretation of the references to colour in this figure legend, the reader is referred to the web version of this article.)

It should be noted that one run was excluded for the evaluation of the amount of fines due to being an outlier, as it fell outside 4 standard deviation according to the residual normal probability, deviating also in the observed versus predicted plot. The effect of screw configuration, such as number of KEs was reduced or dampened by the presence of TMEs at the outlet of the screws. Vercruysse et al. reported less amount of fines with the use of TME in the screw configuration, suggesting that the effect of the number of KEs is less pronounced (Vercruysse et al., 2015a). The GSD is depicted as density distribution (q3) and cumulative distribution (Q3) in Fig. 5 for both formulations obtained under center point process conditions. A close-to monomodal GSD was obtained at the center point for lactose-MCC. In contrast, the utilization of TME resulted in a broader GSD, higher densification and generally larger granules.

The x50 value, which represented the granule size, showed a significant impact of the stagger angle during the granulation of lactose-MCC. The x50 increased with decreased stagger angle (Fig. 4A). The friability of the granules was reduced, in addition to the L/S ratio, by applying thicker KEs. The use of thicker KEs increased the kneading zone and thus improved liquid distribution and granule growth. In both formulations, the thickness did not significantly influence granule size. The friability of the IBU granules was additionally, to the increased L/S ratio, significant negatively affected by the interaction between the number of KEs and thickness, although the factors themselves were not significantly influential. A contour plot is attached in the supplement (Fig. S4). With an increasing number and thickness of KEs, the friability decreased. Further decrease in friability were obtained by using thicker KEs (> 2.8 mm) and fewer KEs.

**Fig. 6:**
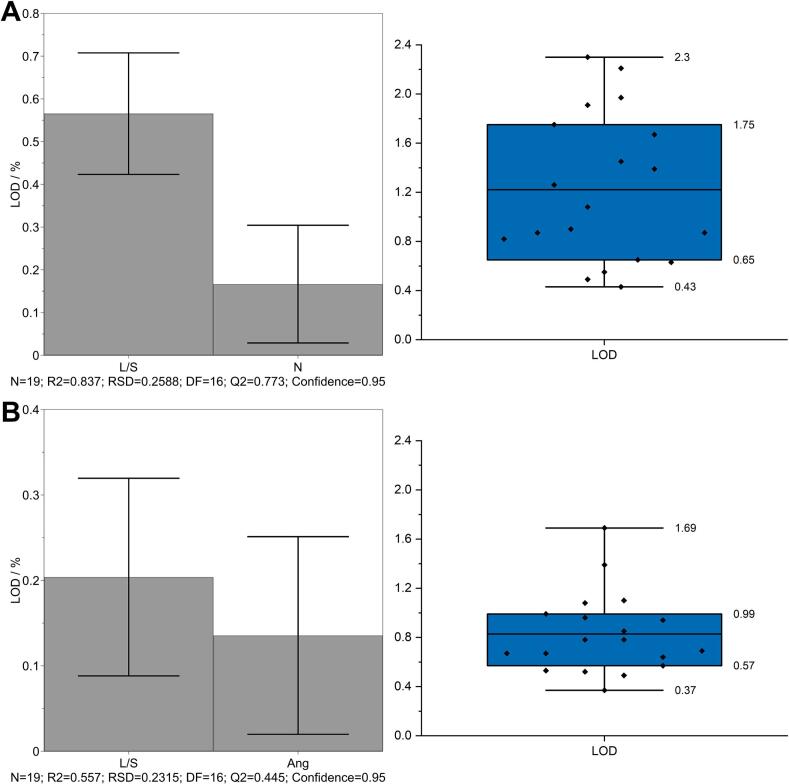
Coefficient plot for LOD (left) as mean coefficient ± 95% confidence interval and box- plot (right) for LOD obtained during granulation of lactose-MCC (A) and IBU (B) formulation. The coefficient constants are 1.22% and 0.83% (B).

The granules from both formulations were dried under different drying conditions. Fig. 6 illustrates the coefficient plots and box-whisker plots for both formulations. LOD increased with a higher L/S ratio and number of KEs (Fig. 6A). With a higher L/S ratio, more water was available for wetting, and with more KEs, the mixing was enhanced, resulting in less porous granules and thus slower drying. The LOD of the IBU granules also increased with increased L/S ratio and stagger angle of KEs, which improved the mixing of the powder with the granulation liquid (Fig. 6B). The ranges for LOD were 0.43% to 2.30% for lactose-MCC and 0.37% to 1.69% for the IBU formulations Generally, higher LODs were obtained for the lactose-MCC granules although a higher drying temperature was applied due to the high water absorption capacity of MCC. The coefficient of the L/S ratio was approximately three times higher for the DoE with lactose-MCC. It should be noted that the factor range of the L/S ratio applied during the DoE with lactose-MCC was 2.5 times broader, resulting in a larger coefficient.

#### 3.1.3 Tablet attributes

**Fig. 7:**
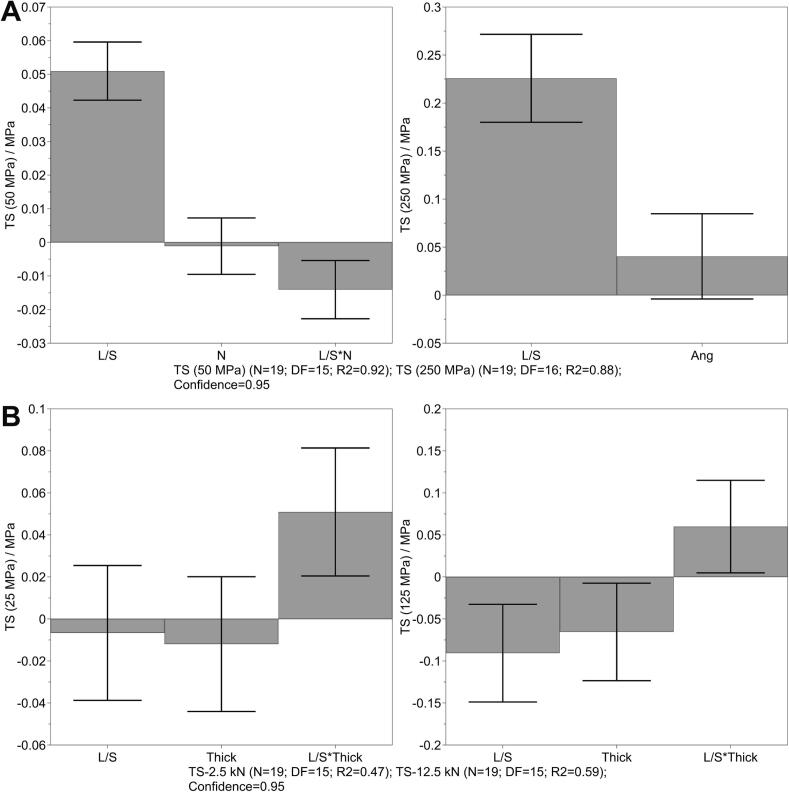
Coefficient plot for TS at lowest (left) and highest (right) compression pressure during granulation and tableting of lactose-MCC (A) and IBU (B) formulation, mean coefficient ± 95% confidence interval. The coefficient constants are 0.47 MPa (A) and 1.36 MPa (B) for TS at lowest compression pressure and 3.42 MPa (A) and 4.20 MPa (B) for TS at highest compression pressure.

The effects of various factors and their interaction were investigated during the down-processing tableting. The obtained values for each run are listed in the Supplement (Tables S4 and S5). Fig. 7 displays the coefficient plots after model optimization for TS obtained at the lowest and highest compression pressure. TS was influenced by the L/S ratio for tableting of lactose-MCC granules at all applied compression pressures. By comparing the coefficients obtained at low and high compression pressures, a higher value indicates a greater effect of L/S ratio at higher compression pressures. Stronger tablets with higher TS were obtained at higher L/S ratio, which might increase the disintegration time. At 50 and 100 MPa compression pressures, the TS was also negatively affected by the interaction between of L/S ratio and number of KEs (Fig. 7A). TS decreased with the use of fewer KEs and higher L/S ratio. During tableting of IBU granules, the TS was significantly influenced by the interaction between L/S ratio and thickness at lower compression pressures. At higher compression pressures (≥ 75 MPa), the TS increased significantly with decreased L/S ratio and thickness. Djuric and Kleinebudde, as well as Vercruysse et al., reported that the usage of less KEs resulted in granules with lower densification thus higher TS (Djuric and Kleinebudde, 2008; Vercruysse et al., 2012). For the IBU granules, the thickness of the KE had a negative influence, especially at high compression pressure, where this factor was significant. The increase in thickness resulted in longer KE-zone, consequently denser granules and lower TS, which corresponded with the findings from the literature.

**Fig. 8:**
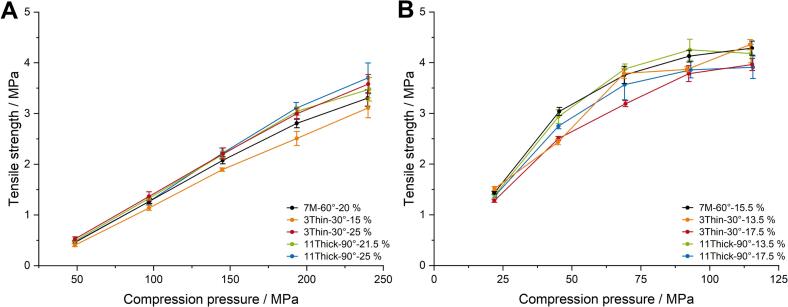
Tabletability profiles obtained at different screw configuration from the DoEs during tableting of lactose-MCC (A) with *n* = 10 and IBU (B) granules with *n* = 6, $\bar{x}\pm s$.

The tabletability of both formulations was exemplary investigated where granules were produced with all factors at both low and high levels, as shown for both L/S ratios and the center point in Fig. 8. The tabletability plot of lactose-MCC granules showed a linear relationship between compression pressure and TS for the applied compression pressures. With higher compression pressure, the TS increased (Fig. 8A). In comparison IBU tablets, less compression pressure was required to obtain tablets with similar TS. The TS increased exponential by applying higher compression pressures (Fig. 8B) without capping of the tablets. This indicates that the granules of the IBU formulation had high compressibility, as less pressure is required for densification. This is shown more clearly in the following section 3.2.2*. Tablet attributes* (Fig. 11B). The tablets obtained from the different granules from the DoE were mostly affected by the L/S ratio thus the moisture content of the granules. Higher moisture content led to improved bonding between the particles. In total, the LOD was below 2.5% for lactose-MCC granules and 2% for IBU granules. Larger differences in TS were obtained by applying higher compression pressures. Tableting of IBU granules showed the opposite effect of L/S ratio on the TS.

### 3.2 Influence of a variation in kneading zone

#### 3.2.1 Granules attributes

**Fig. 9:**
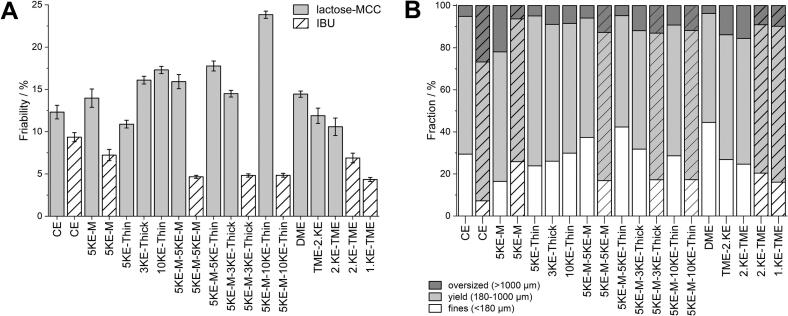
Friability of the yield fraction (A) with *n* = 3 and fraction of fines, yield and oversized (B) of lactose-MCC as empty columns and IBU granules displayed as lined columns, $\bar{x}\pm s$.

The influence of the KE-zone on friability and the fraction of fines, yield and oversized is displayed in Fig. 9. The columns with lines represent values obtained with IBU formulation. Granules produced from the IBU formulation exhibited greater mechanical stress across all configurations, as indicated by friability below 10% in all cases (Fig. 9A), in contrast to lactose-MCC. This observation was confirmed by the tableting results of IBU granules in the DoE study, where less compression pressure was required to achieve tablets with a TS above 2 MPa. Thus, indicating higher densification of the IBU powder during granulation. Comparing the friability values for IBU granules prepared under different configurations, using only CE resulted in the highest friability whereas the application of KE-zone independent of thickness and number of zones led to decreased friability. These granules also yielded the highest fraction of oversized and lowest fraction of fine granules (Fig. 9B) indicating loosely bound and porous granules. Applying TME after the second KE-zone yielded lower friability compared to the granules produced with only CEs. In contrast, the friability of lactose-MCC granules exceeded 10% for all tested configurations. Highest friability with 23.8% was obtained for the granules produced with 10 thin KEs in the second KE-zone. It seems that the granules were less densified and thus consisted of higher porosity which are not resistant against mechanical stress and therefore resulted in high friability. The highest amount of fines was obtained during granulation with the 5KE-M-5KE-thin configuration, which also resulted in high granule friability. In this KE-zone, ready-to-use block of 1D was applied for the second KE-zone, consisting of higher distance between the KEs. Consequently, this configuration led to less densification and lower liquid distribution, thus less granule growth. A high amount of fines was also obtained during granulation with DME, indicating the destruction of oversized granules, which has already reported in the literature (Djuric and Kleinebudde, 2008; Kashani Rahimi et al., 2020). Consequently, the application of at least one KE-zone during the granulation of IBU resulted in less friable granules and a lower amount of fines and oversized granules. In comparison, KE-zone application during the granulation of lactose-MCC showed a lower effect, with only slightly friability. The usage of DME or TME reduced the friability.

**Figure 10:**
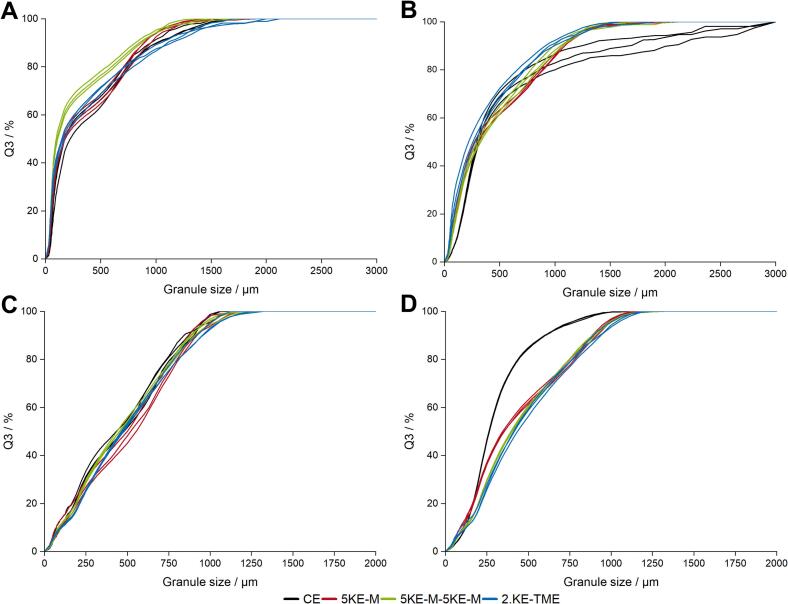
Cumulative GSD of lactose-MCC untreated granules (A), yield fraction (C) and of IBU untreated (B) and yield fraction (D) with n = 3, $\bar{x}\pm s$.

Fig. 10 shows the GSD of the single measurements obtained using only CEs, one or two KE-zones, and also when TME was applied directly after granulation (A, B) as well as for the fractions (C, D). The lactose-MCC granules produced with only CE or TME consisted of larger granules, whereas the use of KE-zones led to smaller granules (Fig. 10A). In contrast, the IBU granules manufactured with only CE were clearly larger than the granules produced with KEs (Fig. 10B). The implementation of a second KE-zone or additional TME in the screw configuration resulted in smaller granules. The difference between with or without KE-zones was evident after fractionation for the IBU granules (Fig. 10D). Thus, the fraction of IBU granules produced with only CE consisted of smaller granules compared to the other configurations. For the lactose-MCC granules, the GSD of the fractions using a was comparable (Fig. 10C).

#### 3.2.2 Tablet attributes

**Figure 11:**
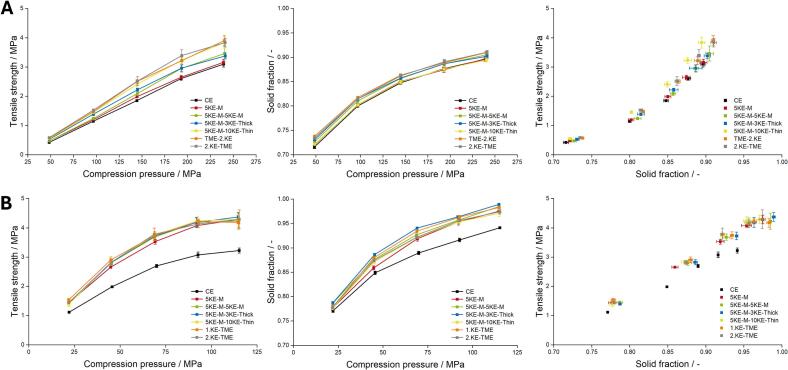
Tabletability (left), compressibility (middle) and compactability (right) profiles obtained of tableting lactose-MCC granules (A) with n = 10 and IBU (B) with n = 6, $\bar{x}\pm s$.

The tabletability, compressibility and compactibility profiles of the granules produced at different screw configuration are shown in Fig. 11. The tabletability profile of lactose-MCC granules showed differences depending on whether the granules were produced using only CE or one KE-zone, two KE-zones or two KE-zones with additional TME. The second KE-zone or TME resulted in higher TS when applying the same compression pressure. The use of a second KE-zone, especially with 10 thin KEs in the second zone, or TME showed comparably high tabletability. By comparing the friability of these granules, the granules produced using 5-KE-M-10KE-Thin as the screw configuration had the highest friability, while the granules produced with TME had the lowest friability (Fig. 9A). The high friability might indicate increased porosity of the granules, leading to higher densification and thus higher TS. Conversely, the use of TME, which increases the residence time due to less conveying capacity, might enhance the liquid distribution, producing less friable and stronger granules, resulting in increased TS. A comparison of the GSD of the yield fractions used for tableting showed no difference for lactose-MCC granules. In Fig. 11B, the tableting of IBU granules indicated a distinct difference on the utilization of KEs. The use of only CE resulted in smaller granules in the yield fraction compared to the other configurations (Fig. 10D). Consequently, when applying the same compression pressure, the tablets yielded a smaller TS compared to the other granules. The granules produced using at least one KE-zone were broken during compression, resulting in new contact surfaces, which improved the interparticular bonding thus led to increased TS. As Yu et al. reported, an increased number of KE improved liquid distribution, which might explain why at least one mixing zone is necessary (Yu et al., 2014). The comparison of the tabletability profiles showed that the tableting of lactose-MCC exhibited a linear relationship between the applied compression pressure and the resulting TS, whereas IBU showed an exponential dependency, reaching a plateau. The IBU granules were highly densified at lower compression pressures, resulting in tablets with high solid fraction (Fig. 11B). By applying a compression pressure of 125 MPa, the granules produced with KEs resulted in a solid fraction up to 100%. In comparison to lactose-MCC, a solid fraction between 85% and 87% was achieved by applying 100 to 150 MPa.

## 4 Conclusion

The influence of the screw configuration in TSG was studied systematically using a DoE approach for two different formulations. The DoE showed that L/S ratio is the most influential parameter in the chosen factor space. Increasing L/S ratio resulted in fewer fines, larger granule size and reduced granule friability. Larger KE thickness resulted in denser granules with lower TS. Additionally, an increase in the number of KEs, angle or thickness led to higher torque and increased energy input, while also increasing the residence time of the granules, potentially improving liquid distribution and granule growth. The use of TME, with its lack of conveying capacity, resulted in a high fraction of oversized granules due to increased residence time and granule growth in the IBU formulation. Therefore, TME should not be positioned at the outlet of the screw configuration without a subsequent milling step. Tabletability profiles indicated a linear relationship between compression pressure and TS for lactose-MCC tablets, with increased compression pressure leading to higher TS. IBU granules exhibited high tabletability, requiring less pressure for tableting, with an initial increase in TS followed by reaching a plateau at higher pressure. Tableting at a compression pressure of 50 MPa yielded TSs of approximately 2.5 MPa for IBU granules and 0.5 MPa for lactose-MCC.

Finally, the systematic investigation of the KE-zone indicated that the screw configuration has a minimal impact on both granules and tablets. At least one KE-zone, independent of its type, is required to achieve granules of low friability and tablets with sufficient strength. The GSD of the lactose-MCC granules showed larger granules when only CEs or TME were used. In comparison, IBU granules exhibited an even greater difference between the KE-zone and only CE, containing a broader GSD and larger granules when only CEs were used. Thus, tableting of granules with a hydrophobic formulation requires at least one KE-zone to improve the liquid distribution and enhance granule and tablet quality, particularly in terms of friability and TS. In cases where granulation serves as an intermediate step requiring further tableting, the influence of screw configuration or KE zone is less critical. Thus, the results of this study provide further support for existing findings regarding the impact of screw elements.

## CRediT authorship contribution statement

**Katharina Kiricenko:** Writing – original draft, Visualization, Methodology, Investigation, Formal analysis, Conceptualization. **Robin Meier:** Writing – review & editing, Methodology, Conceptualization. **Peter Kleinebudde:** Writing – review & editing, Supervision, Methodology, Conceptualization.

## Declaration of competing interest

The authors declare that Katharina Kiricenko and Peter Kleinebudde have no financial interests. Robin Meier is employed by L.B. Bohle Maschinen und Verfahren GmbH.

## Data Availability

Data will be made available on request.

## Appendix A Supplementary data

Supplementary material Image 1
