# Supplementary material for: Systematic investigation of the impact of screw elements in continuous wet granulation
Source: Int J Pharm X. 2024 Aug 8;8:100273. doi: 10.1016/j.ijpx.2024.100273 (PMC11357779; doi:10.1016/j.ijpx.2024.100273)
Supplement: Supplementary file 1 — Supplementary material [file mmc1.pdf]

Systematic investigation of the impact of screw elements in continuous wet granulation

Katharina Kiricenko[a], Robin Meier[b], Peter Kleinebudde[a]*

Supplement

Table S1: Setup of DoE for the lactose-MCC and IBU formulations.

Experiment N°	L/S / % (Lactose-MCC)	L/S / % (IBU)	Kneading block		
			Number KE	Angle / °	KE thickness / mm
1	15	13.5	3	30	1.4
2	25	17.5	3	30	1.4
3	15	13.5	11	30	1.4
4	25	17.5	11	30	1.4
5	15	13.5	3	90	1.4
6	25	17.5	3	90	1.4
7	15	13.5	11	90	1.4
8	25	17.5	11	90	1.4
9	15	13.5	3	30	5.4
10	25	17.5	3	30	5.4
11	15	13.5	11	30	5.4
12	25	17.5	11	30	5.4
13	15	13.5	3	90	5.4
14	25	17.5	3	90	5.4
15	21.5	13.5	11	90	5.4
16	25	17.5	11	90	5.4
17	20	15.5	7	60	2.8
18	20	15.5	7	60	2.8
19	20	15.5	7	60	2.8

Table S2: Obtained responses regarding process and granules of DoE using lactose-MCC, $\bar{x} \pm s$.

N°	MRT / s	Torque / Nm	Fines / %	x50 / µm	Friability / %	LOD / %
1	37.9 ± 6.1	4.4 ± 0.11	34.6	99.2 ± 2.7	41.6 ± 2.0	0.72 ± 0.16
2	37.8 ± 1.0	10.1 ± 0.28	32.4	72.0 ± 1.4	16.2 ± 0.5	0.62 ± 0.10
3	30.8 ± 1.7	5.0 ± 0.12	19.7	126.8 ± 7.7	40.8 ± 1.2	0.67 ± 0.12
4	44.2 ± 1.3	22.7 ± 0.56	25.9	117.1 ± 17.5	12.2 ± 0.5	1.19 ± 0.24
5	37.0 ± 3.1	5.8 ± 0.12	39.0	63.8 ± 1.4	38.2 ± 0.8	0.58 ± 0.09
6	36.1 ± 0.7	10.5 ± 0.41	36.1	67.0 ± 4.7	23.9 ± 0.8	0.55 ± 0.14
7	41.7 ± 1.1	17.6 ± 0.47	23.9	76.6 ± 5.6	21.6 ± 0.8	0.68 ± 0.12
8	65.6 ± 1.7	26.5 ± 0.66	21.9	156.7 ± 15.6	11.5 ± 0.1	1.55 ± 0.17
9	52.6 ± 4.6	6.1 ± 0.08	15.6	352.2 ± 53.3	10.0 ± 0.4	1.38 ± 0.13
10	51.1 ± 2.0	7.8 ± 0.16	18.1	324.2 ± 18.4	8.2 ± 0.4	1.91 ± 0.08
11	39.8 ± 3.7	6.0 ± 0.17	20.8	329.8 ± 21.7	8.5 ± 0.2	2.21 ± 0.29
12	49.0 ± 2.3	13.2 ± 0.30	18.9	242.2 ± 27.2	7.9 ± 0.4	2.20 ± 0.28
13	48.6 ± 4.3	7.0 ± 0.19	19.9	247.2 ± 9.1	8.4 ± 0.5	1.61 ± 0.22
14	48.7 ± 5.6	8.1 ± 0.24	18.0	295.7 ± 14.6	7.2 ± 0.5	1.80 ± 0.37
15	55.0 ± 2.9	11.0 ± 0.30	37.8*	157.0 ± 16.9	11.6 ± 0.3	1.66 ± 0.22
16	65.4 ± 2.5	22.1 ± 0.42	20.5	302.7 ± 16.8	6.1 ± 1.1	1.97 ± 0.17
17	42.8 ± 2.3	6.4 ± 0.13	23.9	114.7 ± 3.6	22.4 ± 0.8	0.78 ± 0.09
18	42.6 ± 1.6	6.4 ± 0.12	23.1	109.8 ± 10.4	19.9 ± 1.4	0.88 ± 0.10
19	44.2 ± 1.7	6.5 ± 0.12	24.5	116.5 ± 17.0	19.3 ± 0.7	0.95 ± 0.23

*Excluded run during evaluation of the response amount of fines.

Table S3: Obtained responses regarding process and granules of DoE using IBU, $\bar{x} \pm s$.

N°	MRT / s	Torque / Nm	Fines / %	x50 / µm	Friability / %	LOD / %
1	63.1 ± 5.4	6.4 ± 0.22	9.7	686.8 ± 205.4	9.2 ± 0.70	0.37 ± 0.04
2	62.4 ± 2.1	4.7 ± 0.15	3.4	1403.8 ± 165.1	4.1 ± 0.28	1.08 ± 0.60
3	47.6 ± 2.0	6.8 ± 0.23	9.8	727.4 ± 209.3	8.2 ± 0.49	0.49 ± 0.23
4	36.6 ± 1.2	6.5 ± 0.29	1.9	1700.4 ± 170.3	4.2 ± 0.16	0.52 ± 0.23
5	53.8 ± 13.5	7.2 ± 0.38	12.8	457.9 ± 157.8	11.3 ± 0.36	0.64 ± 0.16
6	36.2 ± 0.5	5.5 ± 0.37	3.4	1553.9 ± 279.0	4.3 ± 0.40	1.69 ± 0.06
7	45.2 ± 3.5	14.6 ± 0.30	15.9	485.6 ± 180.9	7.4 ± 0.46	0.57 ± 0.37
8	52.8 ± 6.8	11.3 ± 0.50	2.4	1820.9 ± 137.6	4.0 ± 0.59	1.39 ± 0.50
9	43.2 ± 1.8	12.4 ± 0.27	14.2	796.7 ± 126.6	6.0 ± 1.11	0.85 ± 0.09
10	46.8 ± 1.1	9.7 ± 0.41	2.9	1471.4 ± 187.3	3.5 ± 0.44	0.99 ± 0.09
11	52.4 ± 3.7	21.2 ± 0.38	12.8	659.7 ± 79.3	7.2 ± 0.85	0.53 ± 0.06
12	54.7 ± 2.2	15.4 ± 0.50	3.9	1274.1 ± 271.3	3.6 ± 0.57	0.67 ± 0.23
13	31.4 ± 2.1	12.8 ± 1.28	9.9	818.5 ± 51.5	6.2 ± 0.44	0.69 ± 0.22
14	47.9 ± 4.6	9.6 ± 0.41	2.9	1441.3 ± 156.0	3.5 ± 0.75	0.94 ± 0.21
15	49.9 ± 0.7	25.6 ± 0.39	13.4	553.9 ± 237.7	9.5 ± 0.58	0.78 ± 0.06
16	54.5 ± 1.4	19.3 ± 4.25	4.4	1064.2 ± 105.0	4.7 ± 0.09	1.10 ± 0.13
17	46.9 ± 1.7	9.9 ± 0.41	6.3	1384.5 ± 22.2	5.0 ± 0.59	0.96 ± 0.10
18	44.9 ± 2.2	10.0 ± 0.50	5.6	2082.8 ± 60.9	5.4 ± 0.66	0.67 ± 0.38
19	50.7 ± 2.7	10.0 ± 0.54	5.4	1190.4 ± 78.6	5.9 ± 0.61	0.78 ± 0.07

Table S4: Obtained tablet characteristics for the DoE using lactose-MCC, $\bar{x} \pm s$.

N°	CP / MPa	TS / MPa	Solid fraction / -	N°	CP / MPa	TS / MPa	Solid fraction / -
	48.5 $\pm$ 0.24	0.41 $\pm$ 0.03	0.71		48.5 $\pm$ 0.27	0.51 $\pm$ 0.03	0.71
	97.0 $\pm$ 0.40	1.14 $\pm$ 0.05	0.80		97.1 $\pm$ 0.48	1.34 $\pm$ 0.05	0.79
1	144.8 $\pm$ 0.50	1.89 $\pm$ 0.03	0.85	11	144.7 $\pm$ 0.49	2.29 $\pm$ 0.06	0.85
	193.5 $\pm$ 0.54	2.51 $\pm$ 0.14	0.88		192.7 $\pm$ 0.56	3.20 $\pm$ 0.15	0.88
	240.1 $\pm$ 0.65	3.11 $\pm$ 0.20	0.90		240.1 $\pm$ 1.16	3.61 $\pm$ 0.15	0.90
	48.5 $\pm$ 0.26	0.39 $\pm$ 0.03	0.72		48.5 $\pm$ 0.20	0.52 $\pm$ 0.02	0.71
	96.9 $\pm$ 0.41	1.10 $\pm$ 0.06	0.80		96.7 $\pm$ 0.51	1.36 $\pm$ 0.05	0.80
2	144.6 $\pm$ 0.45	1.82 $\pm$ 0.07	0.84	12	145.1 $\pm$ 0.59	2.26 $\pm$ 0.09	0.85
	193.6 $\pm$ 0.53	2.53 $\pm$ 0.08	0.87		193.6 $\pm$ 0.69	3.12 $\pm$ 0.11	0.88
	239.9 $\pm$ 0.54	3.09 $\pm$ 0.18	0.89		240.9 $\pm$ 0.50	3.53 $\pm$ 0.19	0.90
	48.6 $\pm$ 0.20	0.41 $\pm$ 0.02	0.72		48.7 $\pm$ 0.26	0.55 $\pm$ 0.02	0.71
	96.6 $\pm$ 0.36	1.25 $\pm$ 0.05	0.82		97.2 $\pm$ 0.32	1.40 $\pm$ 0.09	0.79
3	144.5 $\pm$ 0.73	2.06 $\pm$ 0.06	0.84	13	144.7 $\pm$ 0.52	2.33 $\pm$ 0.07	0.84
	193.3 $\pm$ 0.66	2.74 $\pm$ 0.09	0.87		193.8 $\pm$ 0.89	3.20 $\pm$ 0.10	0.87
	239.5 $\pm$ 1.04	3.18 $\pm$ 0.18	0.89		239.9 $\pm$ 0.75	3.73 $\pm$ 0.11	0.90
	48.6 $\pm$ 0.31	0.43 $\pm$ 0.01	0.73		48.7 $\pm$ 0.30	0.53 $\pm$ 0.03	0.71
	96.9 $\pm$ 0.45	1.14 $\pm$ 0.04	0.80		97.2 $\pm$ 0.54	1.43 $\pm$ 0.05	0.80
4	145.0 $\pm$ 0.59	1.85 $\pm$ 0.06	0.85	14	145.0 $\pm$ 0.54	2.35 $\pm$ 0.07	0.85
	194.0 $\pm$ 0.66	2.50 $\pm$ 0.09	0.87		193.3 $\pm$ 0.89	3.17 $\pm$ 0.11	0.88
	240.8 $\pm$ 1.20	3.16 $\pm$ 0.23	0.90		240.4 $\pm$ 0.67	3.59 $\pm$ 0.30	0.90
	48.7 $\pm$ 0.23	0.42 $\pm$ 0.02	0.72		48.6 $\pm$ 0.29	0.51 $\pm$ 0.03	0.72
	97.3 $\pm$ 0.31	1.18 $\pm$ 0.05	0.80		96.9 $\pm$ 0.44	1.33 $\pm$ 0.07	0.81
5	144.6 $\pm$ 0.71	2.02 $\pm$ 0.07	0.85	15	144.9 $\pm$ 0.40	2.27 $\pm$ 0.05	0.85
	193.0 $\pm$ 0.45	2.73 $\pm$ 0.16	0.89		193.1 $\pm$ 0.56	2.98 $\pm$ 0.10	0.89
	240.8 $\pm$ 0.71	3.33 $\pm$ 0.14	0.91		241.1 $\pm$ 0.77	3.67 $\pm$ 0.18	0.90
	48.7 $\pm$ 0.27	0.40 $\pm$ 0.02	0.72		48.4 $\pm$ 0.25	0.47 $\pm$ 0.03	0.71
	96.9 $\pm$ 0.41	1.11 $\pm$ 0.05	0.80		96.9 $\pm$ 0.39	1.26 $\pm$ 0.06	0.80
6	145.0 $\pm$ 0.57	1.94 $\pm$ 0.08	0.85	16	144.7 $\pm$ 0.50	2.21 $\pm$ 0.11	0.85
	193.9 $\pm$ 0.50	2.66 $\pm$ 0.09	0.88		193.0 $\pm$ 0.64	3.11 $\pm$ 0.11	0.88
	239.6 $\pm$ 0.72	3.11 $\pm$ 0.19	0.89		240.0 $\pm$ 0.84	3.71 $\pm$ 0.29	0.90
	48.6 $\pm$ 0.26	0.45 $\pm$ 0.03	0.72		48.6 $\pm$ 0.25	0.46 $\pm$ 0.03	0.71
	97.1 $\pm$ 0.43	1.14 $\pm$ 0.05	0.79		97.2 $\pm$ 0.29	1.23 $\pm$ 0.07	0.80
7	144.9 $\pm$ 0.65	1.95 $\pm$ 0.07	0.84	17	145.2 $\pm$ 0.55	2.07 $\pm$ 0.07	0.86
	193.2 $\pm$ 0.60	2.69 $\pm$ 0.07	0.87		193.5 $\pm$ 0.56	2.92 $\pm$ 0.10	0.89
	240.6 $\pm$ 0.63	3.30 $\pm$ 0.18	0.89		240.5 $\pm$ 0.74	3.59 $\pm$ 0.18	0.91
	48.6 $\pm$ 0.37	0.49 $\pm$ 0.02	0.72		48.6 $\pm$ 0.17	0.46 $\pm$ 0.02	0.71
	96.9 $\pm$ 0.38	1.33 $\pm$ 0.04	0.81		97.3 $\pm$ 0.38	1.27 $\pm$ 0.04	0.80
8	145.1 $\pm$ 0.63	2.19 $\pm$ 0.07	0.86	18	145.1 $\pm$ 0.55	2.08 $\pm$ 0.07	0.84
	193.9 $\pm$ 0.64	3.05 $\pm$ 0.08	0.89		193.5 $\pm$ 0.53	2.81 $\pm$ 0.09	0.87
	240.8 $\pm$ 0.91	3.48 $\pm$ 0.23	0.91		239.7 $\pm$ 0.62	3.31 $\pm$ 0.16	0.89
	48.7 $\pm$ 0.24	0.53 $\pm$ 0.04	0.72		48.6 $\pm$ 0.26	0.45 $\pm$ 0.02	0.71
	97.0 $\pm$ 0.40	1.37 $\pm$ 0.09	0.81		97.2 $\pm$ 0.40	1.19 $\pm$ 0.04	0.79
9	145.4 $\pm$ 0.39	2.21 $\pm$ 0.11	0.86	19	144.7 $\pm$ 0.61	2.35 $\pm$ 0.06	0.86
	193.6 $\pm$ 0.84	3.00 $\pm$ 0.11	0.89		193.2 $\pm$ 0.40	2.89 $\pm$ 0.09	0.87
	240.2 $\pm$ 0.84	3.58 $\pm$ 0.19	0.91		239.9 $\pm$ 0.72	3.45 $\pm$ 0.18	0.90
	48.6 $\pm$ 0.21	0.53 $\pm$ 0.04	0.71				
	96.9 $\pm$ 0.42	1.44 $\pm$ 0.05	0.79				
10	144.5 $\pm$ 0.54	2.41 $\pm$ 0.09	0.85			-	
	193.3 $\pm$ 0.61	3.24 $\pm$ 0.13	0.88				
	239.1 $\pm$ 0.83	3.72 $\pm$ 0.24	0.90				

Table S5: Obtained tablet characteristics for the DoE using IBU, $\bar{x} \pm s$.

N°	CP / MPa	TS / MPa	Solid fraction / -	N°	CP / MPa	TS / MPa	Solid fraction / -
	21.9 ± 0.15	1.52 ± 0.04	0.77		22.1 ± 0.12	1.19 ± 0.05	0.77
	44.9 ± 0.24	2.43 ± 0.05	0.86		45.4 ± 0.27	2.52 ± 0.07	0.86
1	68.9 ± 0.42	3.79 ± 0.10	0.90	11	68.9 ± 0.29	3.61 ± 0.11	0.92
	91.7 ± 0.52	3.87 ± 0.05	0.92		92.5 ± 0.39	3.92 ± 0.34	0.94
	114.6 ± 0.55	4.35 ± 0.10	0.95		114.8 ± 0.61	3.97 ± 0.21	0.97
	21.8 ± 0.12	1.28 ± 0.03	0.78		22.2 ± 0.12	1.34 ± 0.09	0.77
	45.1 ± 0.28	2.50 ± 0.04	0.86		45.4 ± 0.24	2.57 ± 0.07	0.86
2	69.4 ± 0.45	3.19 ± 0.06	0.91	12	69.5 ± 0.30	3.65 ± 0.19	0.91
	92.4 ± 0.53	3.78 ± 0.16	0.92		93.2 ± 0.59	3.97 ± 0.12	0.94
	115.4 ± 0.52	3.96 ± 0.12	0.95		115.0 ± 0.49	4.14 ± 0.12	0.97
	21.9 ± 0.10	1.35 ± 0.04	0.78		22.1 ± 0.08	1.33 ± 0.07	0.78
	44.9 ± 0.28	2.60 ± 0.04	0.86		45.4 ±0.28	2.61 ± 0.09	0.87
3	69.2 ± 0.48	3.79 ± 0.12	0.92	13	68.2 ± 0.23	3.69 ± 0.15	0.92
	92.6 ± 0.38	4.28 ± 0.12	0.94		92.5 ± 0.43	3.94 ± 0.18	0.94
	114.7 ± 0.69	4.57 ± 0.14	0.97		115.0 ± 0.59	4.16 ± 0.36	0.97
	22.0 ± 0.03	1.33 ± 0.02	0.78		22.0 ± 0.13	1.41 ± 0.04	0.77
	45.1 ± 0.33	2.68 ± 0.07	0.86		45.3 ± 0.24	2.52 ± 0.05	0.87
4	69.1 ± 0.47	3.37 ± 0.05	0.91	14	69.2 ± 0.46	3.69 ± 0.11	0.92
	92.5 ± 0.54	3.90 ± 0.06	0.93		93.0 ± 0.50	3.95 ± 0.12	0.95
	119.5 ± 0.61	4.06 ± 0.14	0.95		115.2 ± 0.66	4.13 ± 0.14	0.96
	21.9 ± 0.09	1.38 ± 0.05	0.77		21.9 ± 0.13	1.38 ± 0.07	0.77
	44.6 ± 0.27	3.16 ± 0.08	0.87		45.1 ± 0.25	2.94 ± 0.14	0.86
5	69.1 ± 0.57	3.84 ± 0.10	0.91	15	69.2 ± 0.26	3.88 ± 0.10	0.91
	92.5 ± 0.58	4.21 ± 0.17	0.95		92.8 ± 0.57	4.25 ± 0.21	0.95
	115.0 ± 0.68	4.42 ± 0.16	0.96		114.9 ± 0.36	4.18 ± 0.18	0.96
	22.0 ± 0.12	1.29 ± 0.03	0.77		22.1 ± 0.17	1.38 ± 0.04	0.77
	45.2 ± 0.22	2.53 ± 0.06	0.86		45.1 ± 0.24	2.75 ± 0.05	0.87
6	69.0 ± 0.37	3.48 ± 0.11	0.91	16	69.1 ± 0.16	3.57 ± 0.30	0.92
	92.4 ± 0.50	3.83 ± 0.07	0.93		93.1 ± 0.47	3.86 ± 0.16	0.94
	114.8 ± 0.59	4.21 ± 0.13	0.95		115.6 ± 0.66	3.91 ± 0.22	0.97
	22.0 ± 0.11	1.46 ± 0.05	0.77		22.2 ± 0.08	1.40 ± 0.05	0.78
	45.2 ± 0.24	2.71 ± 0.03	0.86		45.5 ± 0.24	2.48 ± 0.04	0.85
7	69.2 ± 0.33	3.83 ± 0.06	0.92	17	69.1 ± 0.36	3.80 ± 0.08	0.92
	92.7 ± 0.39	4.10 ± 0.26	0.94		92.9 ± 0.46	3.98 ± 0.16	0.94
	114.8 ± 0.36	4.34 ± 0.18	0.97		114.5 ± 0.49	4.32 ± 0.18	0.97
	22.2 ± 0.11	1.32 ± 0.05	0.77		22.1 ± 0.15	1.28 ± 0.03	0.77
	45.4 ± 0.20	2.49 ± 0.06	0.85		45.3 ± 0.23	2.91 ± 0.15	0.86
8	69.3 ± 0.39	3.73 ± 0.07	0.92	18	69.4 ± 0.49	3.71 ± 0.12	0.92
	92.9 ± 0.42	3.87 ± 0.22	0.94		92.7 ± 0.43	3.98 ± 0.08	0.94
	115.1 ± 0.50	4.17 ± 0.16	0.97		115.0 ± 0.43	4.16 ± 0.23	0.96
	22.2 ± 0.12	1.26 ± 0.04	0.77		21.8 ± 0.15	1.44 ± 0.03	0.78
	45.3 ± 0.23	2.86 ± 0.11	0.86		45.4 ± 0.61	3.04 ± 0.08	0.88
9	69.4 ± 0.27	3.65 ± 0.09	0.92	19	69.0 ± 0.31	3.76 ± 0.17	0.92
	92.6 ± 0.46	3.90 ± 0.29	0.94		92.5 ± 0.51	4.13 ± 0.12	0.95
	115.5 ± 0.38	4.30 ± 0.17	0.97		115.3 ± 0.31	4.29 ± 0.14	0.97
	22.0 ± 0.13	1.45 ± 0.07	0.78				
	45.6 ± 0.27	2.31 ± 0.06	0.86				
10	69.2 ± 0.29	3.69 ± 0.08	0.92			-	
	93.1 ± 0.49	3.91 ± 0.06	0.94				
	115.6 ± 0.24	4.22 ± 0.13	0.96				

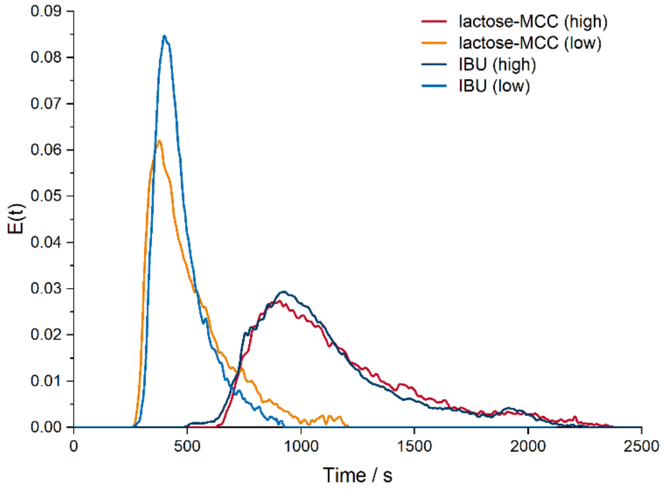

Figure S1: Exemplary RTD curves obtained with all factors at low and high level for both formulations.

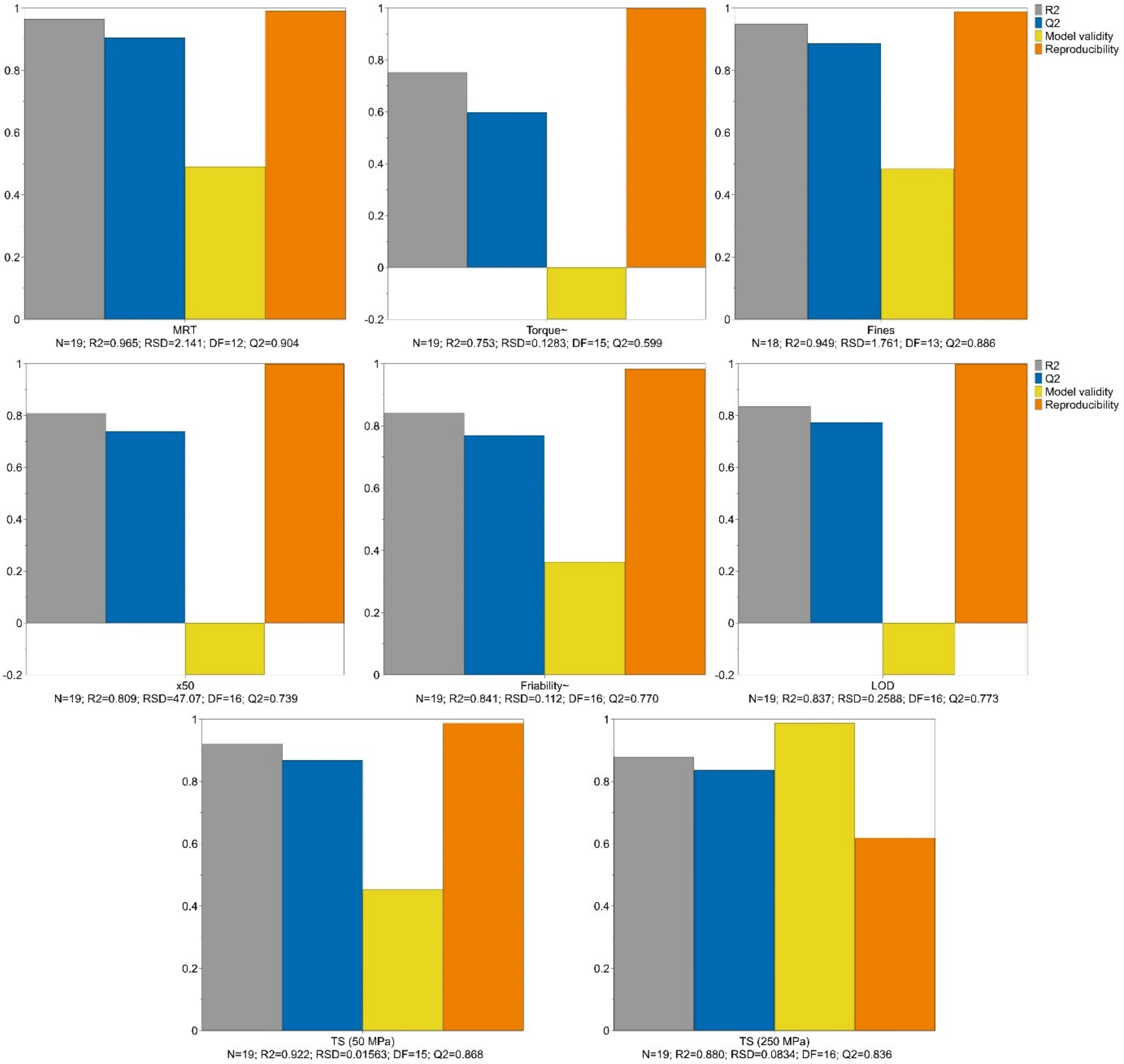

Figure S2: Summary of fit for the models obtained with lactose-MCC formulation.

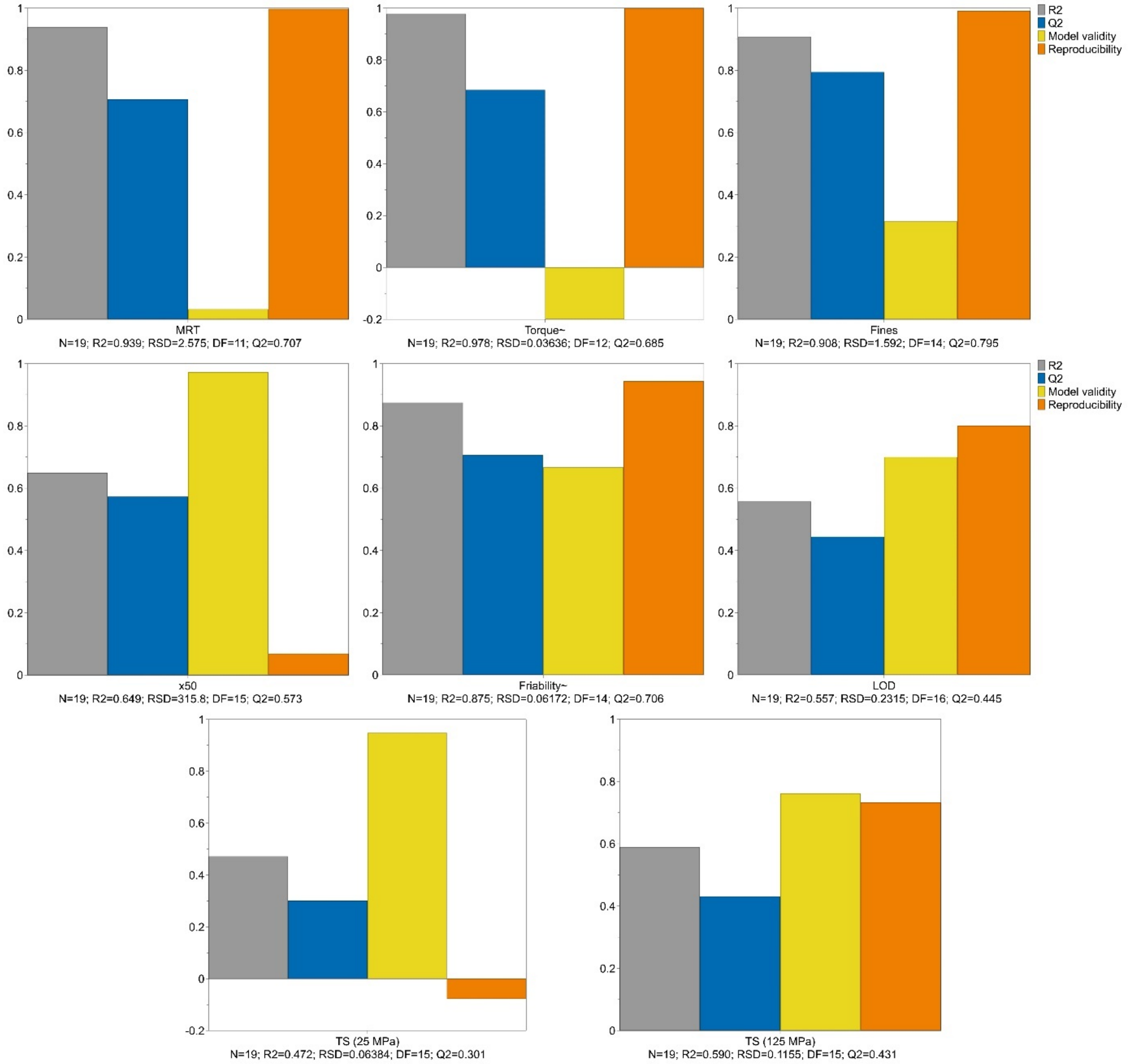

Figure S3: Summary of fit for the models obtained with IBU formulation.

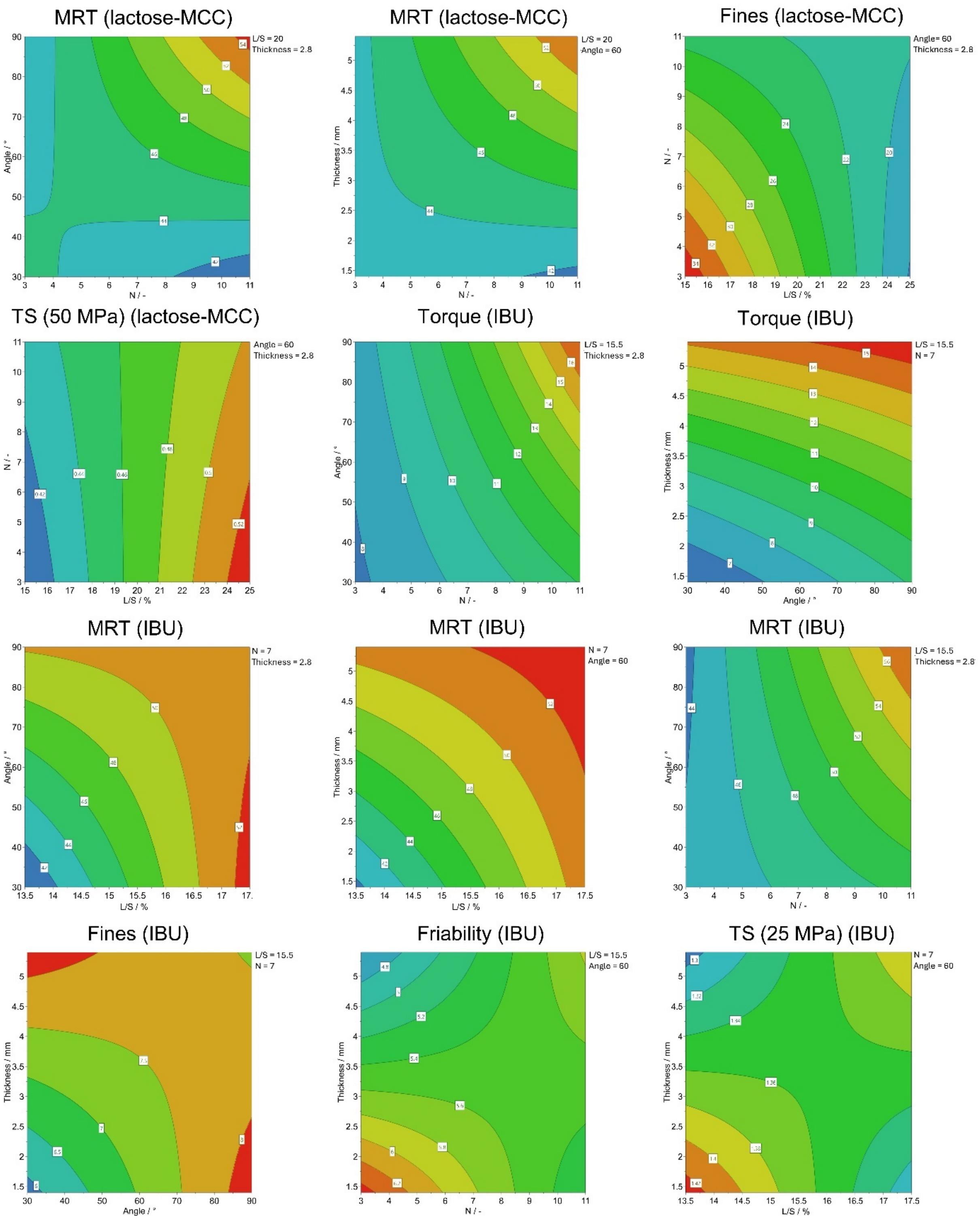

Figure S4: Response contour plots for all significant interactions for both formulations by keeping the other factors at the center point conditions.
